# MicroRNAs in Aneurysmal Subarachnoid Hemorrhage: A Stage-Specific Model Linking Rupture, Vasospasm, and Outcome

**DOI:** 10.3390/biomedicines14061287

**Published:** 2026-06-04

**Authors:** Emre Ozkara, Ebru Erzurumluoglu Gokalp, Ozlem Aykac, Zehra Uysal Kocabas, Sinem Kocagil, Oguz Cilingir, Beyhan Durak Aras, Sevilhan Artan, Atilla Ozcan Ozdemir

**Affiliations:** 1 Department of Neurosurgery, Faculty of Medicine, Eskisehir Osmangazi University, 26140 Eskisehir, Turkey; 2 Department of Medical Genetics, Faculty of Medicine, Eskisehir Osmangazi University, 26140 Eskisehir, Turkey; ebruerzurumluoglu@gmail.com (E.E.G.); sinemkocagil@gmail.com (S.K.); drozi1@gmail.com (O.C.); beyhandurakaras@gmail.com (B.D.A.); sartan26@gmail.com (S.A.); 3 Department of Neurology, Faculty of Medicine, Eskisehir Osmangazi University, 26140 Eskisehir, Turkey; drzlm@yahoo.com (O.A.); drzuysal@hotmail.com (Z.U.K.); atillaozcanozdemir@gmail.com (A.O.O.)

**Keywords:** microRNA, aneurysmal subarachnoid hemorrhage, vasospasm, delayed cerebral ischemia, biomarker, cerebrospinal fluid, rupture, outcome, stage-specific model

## Abstract

Aneurysmal subarachnoid hemorrhage (aSAH) is a life-threatening cerebrovascular condition characterized by a dynamic clinical course spanning distinct pathophysiological stages, including aneurysm rupture, early brain injury (EBI), delayed cerebral vasospasm, and long-term neurological outcome. Despite extensive research, no clinically applicable molecular biomarkers exist to predict disease trajectory across these stages. MicroRNAs (miRNAs), small non-coding RNA molecules detectable in blood and cerebrospinal fluid (CSF), have emerged as promising candidates due to their stability and close association with vascular, inflammatory, and neuronal processes. However, existing studies have largely evaluated miRNAs in isolation, without integrating findings into a unified temporal framework. This review provides a structured, translational synthesis of miRNA dynamics in aSAH and proposes a stage-specific conceptual model integrating prospective clinical evidence with the broader literature. Dual-biofluid profiling has identified miR-29a, miR-200a-3p, and miR-451a as robust rupture-associated biomarkers, with distinct compartment-specific expression patterns. CSF-based profiling has demonstrated that miR-221-3p, miR-9-3p, and miR-183-5p predict vasospasm within 24 h of hemorrhage, while miR-24 and miR-21-5p correlate with disease severity and poor outcome. Integrating these findings with the broader literature, we categorize miRNA signatures across four stages: rupture discrimination, early brain injury, vasospasm prediction, and outcome stratification. This stage-specific framework highlights the biological continuum linking endothelial injury, vascular dysfunction, and secondary brain damage. The proposed model provides a foundation for multi-marker biomarker development, prospective validation studies, and future precision medicine strategies in aSAH.

## 1. Introduction

Aneurysmal subarachnoid hemorrhage (aSAH) is a severe form of hemorrhagic stroke associated with high mortality and long-term neurological disability, despite representing a relatively small proportion of total stroke burden [1]. Advances in neurovascular imaging and treatment strategies have improved early management; however, nearly one-third of patients still die during initial hospitalization, and many survivors experience significant functional impairment [2]. The condition most commonly results from rupture of an intracranial aneurysm (IA), a focal weakening and dilation of the arterial wall [3].

Despite extensive research, no clinically validated molecular biomarkers currently exist to predict stage-specific disease progression in aSAH. The clinical course is not defined solely by the initial hemorrhage but evolves through distinct pathophysiological stages, each associated with different biological mechanisms and therapeutic implications. Early brain injury (EBI), occurring within the first 72 h, is characterized by acute intracranial pressure elevation, oxidative stress, neuroinflammation, and blood–brain barrier disruption [4]. This phase is followed by delayed cerebral vasospasm and delayed cerebral ischemia (DCI), typically developing between days 3 and 10, which remain major contributors to morbidity and poor neurological outcome [5,6].

Given this dynamic and time-dependent disease trajectory, there is a growing need for biomarkers capable of reflecting the evolving biological state of aSAH across its clinical stages. MicroRNAs (miRNAs), small non-coding RNA molecules that regulate gene expression at the post-transcriptional level, have emerged as promising candidates in this context [7,8]. These molecules are detectable in extracellular biofluids such as blood and cerebrospinal fluid (CSF), where they exhibit high stability due to protection by protein complexes or extracellular vesicles [9]. Their expression profiles are closely linked to vascular injury, inflammatory processes, and neuronal damage, making them suitable candidates for biomarker development in cerebrovascular diseases.

Previous studies have investigated miRNA expression patterns at individual stages of aSAH, including aneurysm rupture, vasospasm, and clinical outcome. However, these findings have largely been evaluated in isolation, without integration into a unified temporal or pathophysiological framework. As a result, the potential of miRNAs as stage-specific biomarkers remains insufficiently explored.

In this review, we provide a structured translational synthesis of miRNA dynamics in aSAH and propose a stage-specific conceptual model integrating existing literature with prospective clinical datasets. We categorize miRNA signatures across four stages: (I) aneurysmal rupture and aSAH discrimination, (II) early brain injury, (III) vasospasm and delayed cerebral ischemia, and (IV) clinical severity and neurological outcome. The proposed framework aims to facilitate the development of multi-marker biomarker strategies and to support future precision medicine approaches in aSAH.

## 2. MicroRNAs as Biomarkers: General Principles

MicroRNAs (miRNAs) are small, endogenous, non-coding RNA molecules of approximately 18–22 nucleotides that function as key regulators of post-transcriptional gene expression. Their biogenesis involves a multi-step process in which primary miRNA transcripts are processed into precursor miRNAs by the Drosha–DGCR8 complex, exported to the cytoplasm, and further cleaved by Dicer to generate mature miRNA strands. These mature miRNAs are incorporated into the RNA-induced silencing complex (RISC), where they bind to complementary sequences in target messenger RNAs, leading to translational repression or mRNA degradation [10,11]. Through this mechanism, a single miRNA can regulate multiple target genes and influence diverse biological processes, including vascular remodeling, inflammation, apoptosis, and endothelial function.

A key feature that distinguishes miRNAs from other RNA-based biomarkers is their remarkable stability in extracellular environments. Circulating miRNAs are protected from enzymatic degradation through encapsulation within extracellular vesicles or association with RNA-binding proteins such as Argonaute-2 [12,13]. This stability enables reliable detection in biofluids, including serum, plasma, whole blood, and cerebrospinal fluid (CSF). Quantification is most commonly performed using quantitative real-time polymerase chain reaction, although next-generation sequencing and microarray-based approaches are also widely used [14,15].

An important methodological consideration in aSAH research is the selection of the biological compartment for miRNA analysis. Peripheral blood provides a minimally invasive and repeatable sampling source and reflects systemic responses to hemorrhage; however, it may be influenced by confounding factors such as hemolysis, systemic inflammation, and pharmacological interventions [16]. In contrast, CSF is in direct contact with the subarachnoid space and brain parenchyma, offering a more specific representation of local neurovascular processes, including blood–brain barrier disruption, neuroinflammation, and vascular remodeling [17,18].

These observations suggest that blood and CSF miRNA signals are not interchangeable but provide complementary biological information. Indeed, compartment-specific expression patterns have been reported, supporting the integration of multi-biofluid analysis for improved biomarker performance. Collectively, these properties position miRNAs as promising candidates for stage-specific biomarker development in aSAH.

Recent evidence further suggests that exosomal miRNAs may provide greater biological specificity and stability than freely circulating miRNAs, particularly in neurovascular diseases characterized by endothelial dysfunction and inflammatory signaling [19]. Exosome-associated miRNA transport may also represent an active mechanism of intercellular communication during the progression of aSAH, potentially contributing to propagation of vascular injury, neuroinflammation, and secondary brain damage [19,20]. These findings support the concept of compartment-dependent miRNA signaling, in which blood and CSF reflect biologically distinct yet complementary molecular environments.

## 3. Stage I—Aneurysmal Rupture and aSAH Discrimination

### 3.1. Clinical Perspective

Intracranial aneurysms are present in approximately 2–5% of the general population, most of which remain asymptomatic throughout life [21]. However, rupture leads to aSAH, a catastrophic event associated with high mortality and long-term disability [22,23]. Current imaging-based approaches remain insufficient to accurately predict rupture risk, highlighting the need for reliable molecular biomarkers [16,24].

### 3.2. Evidence from Dual-Biofluid Profiling

Ansari et al. [1] evaluated miRNA expression profiles in blood and CSF samples obtained from patients with ruptured aneurysms, unruptured aneurysms, and healthy controls in a dual-biofluid design. Several miRNAs demonstrated significant differential expression between groups. In blood, miR-29a, miR-200a-3p, and miR-451a were significantly upregulated, showing strong diagnostic performance. In CSF, miR-126, miR-146a-5p, miR-200a-3p, and miR-451a were upregulated, whereas miR-29a and miR-27b-3p were downregulated. Notably, miR-29a, miR-200a-3p, and miR-451a showed consistent discriminative ability across both biofluids.

### 3.3. Biological Interpretation of Key miRNAs

The miR-29 family regulates extracellular matrix components, including collagen and elastin, which are essential for vascular wall integrity [6,25]. Increased circulating miR-29a levels have been associated with aneurysm progression and rupture, while reduced CSF levels may reflect local extracellular matrix degradation. Mechanistically, the divergent compartment-specific behavior of miR-29a likely reflects two distinct biological processes: in the systemic compartment, upregulation corresponds to a generalized vascular stress response and increased extracellular release from injured aneurysm wall tissue, whereas in the cerebrospinal fluid compartment, the relative reduction is consistent with relative local consumption and sequestration through interaction with abundant pro-fibrotic mRNA targets (including COL1A1, COL3A1, and ELN) released during extracellular matrix remodeling at the rupture site [6,25]. This dual behavior is biologically informative rather than contradictory and underscores the value of paired blood–CSF profiling. miR-200a-3p plays a central role in endothelial function and vascular permeability by targeting junctional proteins, thereby contributing to vascular instability [26,27]. Its consistent upregulation across biofluids suggests a direct association with endothelial disruption during rupture. miR-451a is associated with hemolysis and oxidative stress, reflecting red blood cell breakdown and the inflammatory microenvironment following hemorrhage [28,29]. miR-126, an endothelial-enriched miRNA, showed marked elevation in CSF, indicating endothelial injury and vascular dysfunction [23,30].

### 3.4. Summary of Stage I

Overall, Stage I is characterized by a distinct miRNA signature reflecting vascular disruption, endothelial injury, and extracellular matrix remodeling. The compartment-specific expression patterns observed across blood and CSF highlight the complementary roles of these biofluids in biomarker assessment. Independent profiling efforts using rupture-specific miRNA panels have similarly identified circulating miRNAs that discriminate ruptured from unruptured aneurysms, reinforcing the biological convergence of rupture-associated signals across cohorts [31].

## 4. Stage II—Early Brain Injury

Early brain injury (EBI) occurs within the first 72 h following aneurysmal rupture and represents a critical determinant of subsequent disease progression [7,32]. It involves blood–brain barrier disruption, neuroinflammation, oxidative stress, and neuronal apoptosis. Early CSF profiling has demonstrated that miR-126, miR-29a, and miR-27b-3p are significantly downregulated within the first 24 h, while miR-221-3p and miR-183-5p are upregulated [33]. These findings suggest that miRNA expression changes during EBI reflect acute vascular and inflammatory responses rather than vasospasm-specific mechanisms. Among these, miR-126 appears to be a central marker of endothelial injury. Its sustained suppression from early time points through later stages indicates a continuous endothelial dysfunction axis linking EBI to delayed complications [30,34].

Emerging evidence also suggests that early alterations in miRNAs such as miR-34c may predict delayed cerebral ischemia, indicating that molecular predisposition to vasospasm may already be established during the EBI phase [35,36].

Clinical manifestations during the EBI window also carry independent prognostic weight. Early seizures, observed in a substantial proportion of aSAH patients, have been identified as predictors of poor in-hospital outcome and very early mortality [37]. From a molecular standpoint, this clinical observation aligns conceptually with the upregulation of miR-9-3p—a miRNA involved in neuronal excitability and synaptic plasticity—providing a potential mechanistic substrate linking EBI-phase neuronal hyperactivation to subsequent neurological deterioration. Systematic capture of early seizure events was not part of the protocols of the studies discussed here, and prospective integration of electroclinical data with miRNA profiling represents a relevant avenue for future biomarker-clinical correlation cohorts. More broadly, these observations support the concept that early electrophysiological instability and molecular neuroinflammatory activation may represent biologically interconnected processes during the EBI phase.

## 5. Stage III—Vasospasm and Delayed Cerebral Ischemia Prediction

Delayed cerebral vasospasm and its ischemic consequence, delayed cerebral ischemia (DCI), represent the most clinically significant secondary complications of aSAH, typically occurring between days 3 and 10 after hemorrhage [38,39]. Despite advances in monitoring and management, prediction remains largely reactive, and no validated early molecular biomarkers are currently available [40,41]. In a prospective CSF-based study, Ozkara et al. [33] evaluated miRNA expression at days 1 and 5 following hemorrhage in patients with and without vasospasm. The two sampling timepoints were selected to bracket the clinically relevant vasospasm window: day 1 lies clearly before the conventional onset of clinical vasospasm and therefore captures a predictive molecular signal, whereas day 5 falls within the typical 3–10-day diagnostic interval and is therefore positioned to reflect early intra-window pathophysiology. Among the analyzed candidates, three miRNAs demonstrated significant early predictive value for vasospasm: miR-221-3p, miR-9-3p, and miR-183-5p.

miR-9-3p exhibited the strongest discriminative performance, with significant elevation in patients who developed vasospasm compared to those who did not. This early increase, detectable within 24 h, suggests a role in neuronal injury and neuroinflammatory priming [3]. Similarly, miR-183-5p was significantly elevated in vasospasm-positive patients and showed a sustained increase over time, correlating with disease severity [42].

miR-221-3p demonstrated a more complex temporal pattern. While generally elevated after hemorrhage, its relative suppression in vasospasm-prone patients during the early phase suggests dysregulated vascular response mechanisms. This miRNA is known to regulate vascular smooth muscle cell (VSMC) proliferation and endothelial function, supporting its role in vasospasm pathophysiology [36,43].

By day 5, sustained suppression of miR-126 emerged as a highly accurate marker of vasospasm-related vascular dysfunction, with strong diagnostic performance [30,44]. In parallel, miR-24 showed increased expression, reflecting its involvement in endothelial nitric oxide synthase and heme oxygenase pathways, both critical for vascular tone regulation [13,45].

Independent studies support these findings. Elevated miR-221-3p and miR-21-5p levels have been associated with DCI in CSF-based analyses [2], while multi-miRNA panels including miR-146a-5p and miR-27b-3p have demonstrated predictive value for vasospasm [36]. Longitudinal CSF profiling across the first 10 days after aSAH has further demonstrated temporal clustering of miRNA expression into distinct early- and late-phase signatures, providing independent support for the temporal stratification proposed here [46]. These converging data reinforce the concept that vasospasm susceptibility is established early and reflected in distinct miRNA signatures. Overall, Stage III is characterized by miRNAs reflecting vascular constriction, endothelial dysfunction, and neuroinflammatory activation, with early predictive signals detectable prior to clinical manifestation. The biological significance of individual miRNAs appears to be highly time-dependent, suggesting that biomarker interpretation without temporal stratification may obscure clinically relevant molecular dynamics.

The clinical impact of DCI is further modulated by its vascular topography. Outside the aSAH setting, infarctions in the posterior cerebral artery territory have been associated with relatively more favorable functional outcomes than middle cerebral artery infarctions [47], reflecting differences in eloquent cortex involvement, collateral supply, and rehabilitative potential. Whether vascular territory–specific miRNA signatures exist following aSAH-related DCI remains unexplored; correlation of CSF or blood miRNA profiles with infarct topography was not systematically addressed in the prospective studies summarized here, and prospective imaging–molecular integration represents an important direction for future investigation.

## 6. Stage IV—Clinical Severity and Neurological Outcome

Neurological outcome following aSAH is determined by a combination of initial injury severity and secondary complications, including EBI and vasospasm [22]. While clinical grading systems such as Hunt–Hess and WFNS scores provide valuable prognostic information, they do not fully capture the underlying molecular processes driving recovery or deterioration.

miRNAs have emerged as potential integrative biomarkers reflecting cumulative brain injury. In the Ozkara et al. cohort [33], miR-24, miR-21-5p, and miR-183-5p showed strong associations with disease severity and functional outcome.

miR-24 demonstrated a mechanistically significant role, as it negatively regulates both endothelial nitric oxide synthase and heme oxygenase-1, thereby promoting vasoconstriction and oxidative stress [13,45]. Elevated levels of miR-24 were associated with severe hemorrhage, higher clinical grades, and poor functional outcome.

miR-21-5p exhibited a severity-dependent expression pattern, with early elevation in patients with severe neurological presentation and sustained increase in those with poor outcome. This miRNA is known to regulate inflammatory and apoptotic pathways, contributing to both protective and pathological processes [2].

miR-183-5p showed consistent correlation across multiple clinical scales, including Fisher grade, Hunt–Hess score, WFNS score, and modified Rankin Scale, indicating its potential as an integrative outcome biomarker [42]. Its sustained elevation suggests a role in ongoing vascular and inflammatory injury.

Additional candidates include miR-17-5p, associated with severe hemorrhage burden, and miR-143-3p/miR-145-5p, reflecting VSMC phenotypic changes [18,48]. Blood-based markers such as miR-502-5p and miR-1297 have also been associated with poor outcomes and may provide accessible prognostic indicators [49,50].

Furthermore, miR-9-3p has been independently linked to poor functional outcome, reinforcing its dual role in both vasospasm prediction and long-term prognosis [3]. Independent serum profiling has identified additional outcome-associated candidates, including miR-132-3p and miR-324-3p, complementing the blood-based miR-502-5p and miR-1297 signatures [51]. Taken together, Stage IV miRNA signatures reflect cumulative vascular, inflammatory, and neuronal injury, offering potential tools for outcome stratification and risk prediction.

Beyond the cerebral compartment, aSAH frequently induces systemic cardiovascular sequelae, including neurogenic stunned myocardium and Takotsubo-like cardiomyopathy, which contribute independently to morbidity and mortality. miRNAs implicated in cardiac remodeling, hypertrophy, fibrosis, and adverse ventricular response—extensively characterized in heart failure and cardiac resynchronization therapy contexts [52]—share regulatory pathways (notably miR-21, miR-29, and miR-126 family members) with the cerebrovascular signatures discussed here. This conceptual overlap raises the possibility that a subset of circulating miRNAs reflects a combined neurocardiac injury axis after severe neurovascular injury, an avenue that merits dedicated investigation in future biofluid and tissue studies.

## 7. The Integrated Stage-Specific Model

### 7.1. Rationale for a Stage-Specific Framework

**Figure 1:**
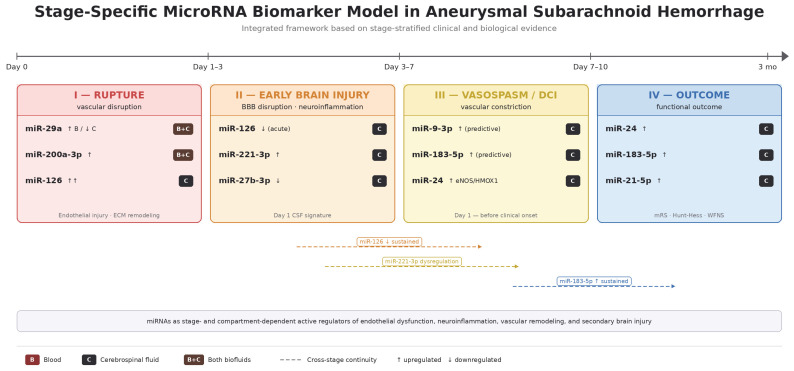
Stage-specific model of microRNA dynamics in aneurysmal subarachnoid hemorrhage. The timeline illustrates four phases: rupture, early brain injury, vasospasm, and outcome. Key miRNAs are mapped according to biofluid source and expression pattern, highlighting temporal and biological continuity across stages.

The stage-specific framework proposed in this review is summarized in Figure 1, which integrates miRNA dynamics across the clinical course of aSAH.

The preceding sections demonstrate that miRNA expression patterns vary significantly across different phases of aSAH, reflecting distinct underlying biological processes. No single miRNA sufficiently captures the full complexity of disease progression. Instead, different miRNAs exhibit peak biological relevance and diagnostic performance at specific stages. This observation supports a stage-specific model in which miRNAs are not considered as isolated biomarkers but as components of a dynamic, temporally structured system. This framework, therefore, positions miRNA behavior in aSAH as fundamentally stage- and compartment-dependent rather than biologically static across the disease course. This framework is supported by prospective clinical datasets [1,33] and corroborated by multiple independent studies [2,36]. Key miRNA candidates across stages are summarized in Table 1.

### 7.2. Stage I—Rupture and aSAH Discrimination

The initial stage is characterized by vascular disruption, endothelial injury, and extracellular matrix remodeling. Key miRNAs include miR-29a, miR-200a-3p, and miR-451a, which demonstrate strong diagnostic performance across both blood and CSF [1]. A defining feature of this stage is the compartment-specific expression pattern of miR-29a, which is upregulated in blood but downregulated in CSF. This finding highlights that systemic and local responses to aneurysm rupture differ and that dual-biofluid analysis provides complementary information.

### 7.3. Stage II—Early Brain Injury

Early brain injury represents a critical transition phase linking initial hemorrhage to subsequent complications. During this stage, suppression of miR-126, miR-29a, and miR-27b-3p reflects endothelial dysfunction and blood–brain barrier disruption [33]. Among these, miR-126 emerges as a central marker of endothelial integrity. Its sustained suppression across timepoints suggests that early vascular injury is not transient but persists into later stages. This supports the concept that early biological events during EBI influence subsequent vasospasm risk.

### 7.4. Stage III—Vasospasm and Delayed Cerebral Ischemia

The vasospasm stage is characterized by vascular constriction, smooth muscle cell remodeling, and neuroinflammatory activation. Early predictive miRNAs include miR-221-3p, miR-9-3p, and miR-183-5p, which demonstrate significant differential expression within 24 h of hemorrhage [33]. These miRNAs represent distinct biological processes: vascular remodeling (miR-221-3p), neuronal injury (miR-9-3p), and oxidative-inflammatory stress (miR-183-5p). At later timepoints, sustained suppression of miR-126 and increased expression of miR-24 reflect ongoing vascular dysfunction and impaired nitric oxide signaling [30,45]. The functional significance of sustained miR-126 suppression in vasospasm pathophysiology is supported by converging mechanistic and clinical evidence: miR-126 directly regulates VCAM-1–mediated endothelial activation and vascular permeability [34], and low circulating miR-126 levels have been associated with aneurysm rupture risk and unfavorable cerebrovascular outcomes in independent cohorts and meta-analytic synthesis [30,44]. These findings support the concept that vasospasm is not an isolated event but a continuation of earlier endothelial injury reflected in a persistent miR-126 deficit.

### 7.5. Stage IV—Clinical Severity and Outcome

The final stage reflects cumulative brain injury and determines functional outcome. Key miRNAs include miR-24, miR-21-5p, and miR-183-5p, which correlate with clinical severity and poor neurological outcome [33]. miR-24 is particularly notable due to its mechanistic role in regulating endothelial nitric oxide synthase and heme oxygenase pathways, linking molecular changes directly to vascular dysfunction [45]. In addition, blood-based miRNAs such as miR-502-5p and miR-1297 provide accessible indicators of systemic injury and outcome risk [49,50], while miR-9-3p has been associated with poor functional recovery independent of clinical grading systems [3]. Supporting evidence from previous studies is summarized in Table 2.

### 7.6. Key Biological Insights

Several important insights emerge from this integrated framework: First, miRNA expression is highly stage-dependent, and biomarker performance is optimized when interpreted within a temporal context. Second, blood and CSF provide complementary information rather than interchangeable signals, as demonstrated by compartment-specific expression patterns such as miR-29a. Third, early endothelial injury, reflected by sustained suppression of miR-126, represents a central axis linking early brain injury to vasospasm development. Fourth, specific miRNAs, particularly miR-24, provide mechanistic insight into disease progression and may represent potential therapeutic targets. Taken together, these patterns support the concept that miRNAs are not merely passive biomarkers but active regulators of endothelial dysfunction, inflammatory amplification, vascular remodeling, and secondary brain injury in aSAH. Mechanistic interpretation of key miRNAs is presented in Table 3.

## 8. Limitations and Future Directions

Several limitations should be acknowledged. The principal empirical studies underlying this framework were based on relatively modest observational cohorts, limiting subgroup analyses and precluding robust multivariable modeling or causal inference; their use of pre-specified candidate miRNA panels rather than unbiased genome-wide profiling also constrains discovery scope. In addition, the use of U6 as a single endogenous reference gene has recognized limitations in extracellular biofluids due to expression variability and sensitivity to pre-analytical conditions [4,56]. IBM SPSS Statistics version 25.0 and GraphPad Prism version 8.0 were used for statistical analyses in the underlying studies. Finally, the broader literature is characterized by substantial methodological heterogeneity—including variation in patient cohorts, biofluid types, sampling timepoints, quantification platforms, and normalization strategies—that limits cross-study comparisons and has prevented formal meta-analysis for most individual miRNAs.

Future research should prioritize large-scale prospective multicenter validation studies enrolling several hundred aSAH patients, collecting both blood and CSF at multiple pre-specified timepoints, and applying blinded molecular analysis with standardized pre-analytical protocols. Multi-miRNA panel development through systematic feature selection—including LASSO regression and machine learning approaches—combined with established clinical predictors such as Fisher grade and Hunt-Hess score, will likely yield superior predictive performance over single biomarkers. Unbiased genome-wide miRNA profiling using next-generation sequencing should be applied to well-characterized aSAH cohorts at each disease stage. Mechanistic investigations are needed to confirm the biological roles of identified miRNAs in human aSAH tissue—particularly the miR-24/eNOS/HMOX1 axis in vasospasm and the miR-126/VCAM-1 axis in BBB disruption. At the translational level, development of point-of-care detection platforms, exploration of miRNA-based therapeutic strategies (including miR-24 inhibition and miR-126 replacement), and integration with multi-omics and artificial intelligence platforms represent the longer-term horizon for clinical implementation. Integration of longitudinal miRNA profiling with radiomics, continuous physiological monitoring, and artificial intelligence-assisted predictive modeling may further improve individualized risk stratification and treatment selection in aSAH.

## 9. Conclusions

Aneurysmal subarachnoid hemorrhage is a phase-dependent disease in which distinct biological processes drive clinical evolution from aneurysm rupture to long-term neurological outcome. The evidence synthesized in this review indicates that circulating microRNAs, measured in blood and cerebrospinal fluid, reflect these stage-specific processes and therefore represent promising candidates for biomarker development.

The principal contribution of this work is the proposal of a stage-specific conceptual framework that organizes miRNA signatures according to their relevance across four major phases: rupture discrimination, early brain injury, vasospasm and delayed cerebral ischemia, and clinical outcome. This framework is supported by prospective clinical datasets [1,33] and integrated with findings from the broader literature, providing a coherent model that links endothelial injury, vascular dysfunction, and secondary brain damage across time.

Several key observations emerge. First, miRNA expression is highly stage-dependent, and biomarker performance is optimized when interpreted within a temporal context. Second, blood and CSF provide complementary, non-redundant biological information, as illustrated by compartment-specific expression patterns such as miR-29a. Third, sustained endothelial injury, reflected by persistent suppression of miR-126, appears to represent a central axis connecting early brain injury to delayed vasospasm. Finally, mechanistically relevant miRNAs, including miR-24, may offer insights not only for prognostic assessment but also for potential therapeutic targeting.

At the translational level, these findings support a multi-marker, stage-adapted biomarker strategy rather than reliance on single candidates. Early blood-based panels may assist in rupture discrimination and initial risk assessment, while CSF-based measurements during the acute phase may improve prediction of vasospasm and disease severity. However, substantial challenges remain before clinical implementation can be achieved.

Future research should prioritize large multicenter longitudinal cohorts integrating blood and CSF profiling across the full disease course, combined with genome-wide miRNA discovery approaches and mechanistic validation studies. Integration of miRNA signatures with imaging biomarkers, multi-omics platforms, and artificial intelligence-assisted predictive models may further improve individualized risk stratification and translational applicability in aSAH.

In conclusion, microRNAs provide a biologically coherent and clinically promising framework for understanding and monitoring the dynamic course of aSAH. The stage-specific model proposed here offers a structured basis for future research and may contribute to the development of precision medicine approaches in this complex disease.

## Figures and Tables

**Table 1:** Stage-Specific MicroRNA Signatures in Aneurysmal Subarachnoid Hemorrhage.

	Key miRNAs	Direction	Biofluid	Biological Role
I—Rupture/aSAH	miR-29a	↑ blood/↓ CSF	B, C	ECM remodeling
	miR-200a-3p	↑	B, C	Endothelial permeability
	miR-451a	↑	B, C	Hemolysis, oxidative stress
	miR-126	↑	B, C	Endothelial injury
II—Early Brain Injury	miR-126	↓	C	BBB disruption
	miR-29a	↓	C	ECM injury
	miR-27b-3p	↓	C	Vascular signaling
	miR-221-3p	↑	C	Inflammatory activation
III—Vasospasm/DCI	miR-221-3p	↓ (early)	C	VSMC remodeling
	miR-9-3p	↑	C	Neuronal injury
	miR-183-5p	↑	C	Oxidative stress
	miR-24	↑ (day 5)	C	eNOS/HMOX1 axis
IV—Severity/Outcome	miR-24	↑	C	Vascular dysfunction
	miR-21-5p	↑	C	Inflammation/apoptosis
	miR-183-5p	↑	C	Severity marker
	miR-502-5p	↑	B	Systemic outcome

Abbreviations: B, blood; C, cerebrospinal fluid; ECM, extracellular matrix; BBB, blood–brain barrier; VSMC, vascular smooth muscle cell; DCI, delayed cerebral ischemia. ↑, upregulated/increased expression; ↓, downregulated/decreased expression.

**Table 2:** Key Studies on MicroRNAs in Aneurysmal Subarachnoid Hemorrhage and Related Stages.

Study	Year	Sample	*n*	Timepoint	Stage Focus	Key miRNAs	Main Finding
Li et al. [53]	2014	Plasma	IA vs. control	Acute	Rupture	miR-16, miR-25	Plasma miRNAs differentiate IA from controls
Su et al. [51]	2015	Serum	22 aSAH	Day 0–3	EBI	miR-132-3p	Early miRNA elevation after aSAH
Powers et al. [46]	2016	CSF	aSAH	Days 1–10	Vasospasm/DCI	multiple	Longitudinal CSF profiling reveals temporal clustering of miRNA expression
Stylli et al. [29]	2017	CSF	19 aSAH	Days 1–18	Vasospasm	miR-451a, miR-27a-3p	CSF miRNA changes linked to vasospasm
Bache et al. [2]	2017	CSF, plasma	40 aSAH	Days 3–7	Vasospasm/DCI	miR-21-5p, miR-221-3p	Elevated in DCI patients
Lopes et al. [28]	2018	Whole blood	27 aSAH	Days 7–10	aSAH	miR-451a	Global miRNA changes after SAH
Sheng et al. [49,50]	2018	Serum	60 aSAH	Day 3	Outcome	miR-502-5p, miR-1297	Associated with poor neurological outcome
Bache et al. [3]	2020	CSF	57 aSAH	Day 3	Outcome	miR-9-3p	Predicts poor functional outcome
Supriya et al. [31]	2020	Plasma	IA cohort	Pre/post rupture	Rupture	panel	Circulating miRNAs distinguish ruptured from unruptured aneurysms
Liao et al. [19]	2023	Exosomes	Review	Various	Biomarkers	exosomal miRNAs	Exosomal miRNAs in aSAH pathogenesis and clinical applications
Ozkara et al. [33]	2025	CSF	40 aSAH	Days 1 & 5	Vasospasm/Outcome	miR-221-3p, miR-9-3p, miR-183-5p	Early vasospasm prediction and severity correlation
Villegas-Gomez et al. [54]	2025	Review	21 studies	Various	Rupture	miR-29a	Most consistent rupture-associated miRNA
Palermo et al. [55]	2025	Blood/CSF	Multiple	Various	Biomarkers	miR-126, miR-200a-3p	Validated liquid biopsy candidates
Shafeeque et al. [20]	2025	CSF exosomes	aSAH	Acute	DCI	exosomal miRNAs	CSF-exosomal miRNAs inform DCI pathophysiology

Abbreviations: aSAH, aneurysmal subarachnoid hemorrhage; CSF, cerebrospinal fluid; DCI, delayed cerebral ischemia; IA, intracranial aneurysm.

**Table 3:** Mechanistic and Translational Interpretation of Key MicroRNAs in Aneurysmal Subarachnoid Hemorrhage.

miRNA	Primary Stage	Main Biological Axis	Direction	Translational Interpretation	Key Evidence
miR-29a	Rupture/EBI	ECM remodeling, vessel wall integrity	↑ blood/↓ CSF	Compartment-specific rupture biomarker; reflects systemic rupture response and local vascular wall remodeling	[1,6,25]
miR-200a-3p	Rupture	Endothelial permeability	↑ blood and CSF	Strong rupture-associated marker; may reflect endothelial disruption and vascular instability	[1,26,27]
miR-451a	Rupture/Severity	Hemolysis, oxidative stress	↑ blood and CSF	Blood-accessible aSAH marker reflecting erythrocyte breakdown and oxidative injury	[1,28,29]
miR-126	Rupture/EBI/Vasospasm	Endothelial integrity, VEGF signaling, BBB homeostasis	↑ at rupture/↓ during EBI–vasospasm	Dual-phase endothelial marker; sustained suppression may link EBI to vasospasm susceptibility	[23,33,34,44]
miR-221-3p	EBI/Vasospasm/Outcome	VSMC remodeling, endothelial dysfunction	Altered; ↓ early in VSP+ vs. VSP−	Complex temporal biomarker; early dysregulation may identify vasospasm-prone patients	[2,33,36]
miR-9-3p	Vasospasm/Outcome	Neuronal injury, neuroinflammation	↑ CSF	Strong early vasospasm predictor; also associated with poor functional outcome	[3,33]
miR-183-5p	Vasospasm/Outcome	Oxidative-inflammatory vascular injury	↑ CSF	Early vasospasm signal; sustained elevation correlates with clinical severity	[33,42]
miR-24	Vasospasm/Outcome	eNOS/HMOX1 axis, vascular tone, oxidative injury	↑ CSF, especially day 5	Mechanistically supported severity marker and potential therapeutic target	[13,33,45]
miR-21-5p	EBI/Outcome	Inflammation, apoptosis, vascular remodeling	↑ CSF	Severity-associated marker; may reflect ongoing inflammatory and apoptotic injury	[2,33]
miR-502-5p	Rupture/Outcome	Systemic injury response	↑ blood	Blood-accessible marker associated with severity and poor outcome	[1,49]
miR-1297	Rupture/Outcome	Systemic injury response	↑ blood	Early serum marker associated with poor neurological outcome	[1,50]
miR-34c	EBI/DCI	BBB disruption, redox dysregulation	↑ plasma	Early blood-accessible DCI predictor; requires validation because current evidence includes preprint data	[35]

Abbreviations: BBB, blood–brain barrier; CSF, cerebrospinal fluid; DCI, delayed cerebral ischemia; EBI, early brain injury; ECM, extracellular matrix; eNOS, endothelial nitric oxide synthase; HMOX1, heme oxygenase-1; VEGF, vascular endothelial growth factor; VSMC, vascular smooth muscle cell; VSP+, vasospasm-positive; VSP−, vasospasm-negative. ↑, upregulated/increased expression; ↓, downregulated/decreased expression.

## Data Availability

No new data were created or analyzed in this study.

## Abbreviations

aSAH	Aneurysmal subarachnoid hemorrhage
BBB	Blood–brain barrier
CSF	Cerebrospinal fluid
DCI	Delayed cerebral ischemia
EBI	Early brain injury
ECM	Extracellular matrix
eNOS	Endothelial nitric oxide synthase
HMOX1	Heme oxygenase-1
IA	Intracranial aneurysm
mRS	Modified Rankin Scale
VSMC	Vascular smooth muscle cell
WFNS	World Federation of Neurosurgical Societies
VSP+	Vasospasm-positive
VSP−	Vasospasm-negative
